# Association of Blood Arsenic Concentrations with Lipid Markers in Uruguayan Adolescents: Exploring Effect Modification by Body Mass Index and Sex

**DOI:** 10.1007/s12403-026-00768-x

**Published:** 2026-04-14

**Authors:** Gauri Desai, Elena I. Queirolo, Teresa Quattrin, Patrick J. Parsons, Christopher D. Palmer, María Inés Beledo, Katarzyna Kordas

**Affiliations:** 1 Department of Epidemiology and Environmental Health, University at Buffalo, Buffalo, NY USA; 2 Department of Neuroscience and Learning, Catholic University of Uruguay, Montevideo, Uruguay; 3 Department of Pediatrics, Jacobs School of Medicine and Biomedical Sciences, University at Buffalo, Buffalo, NY USA; 4 Division of Environmental Health Sciences, Wadsworth Center, New York State Department of Health, Albany, NY USA; 5 Department of Environmental Health Sciences, College of Integrated Health Sciences, University at Albany, Albany, NY USA

**Keywords:** Low-level arsenic, Lipids, Adolescents, Montevideo

## Abstract

Our study examined the association between blood As and total cholesterol (TC), high-density lipoprotein cholesterol (HDL-C), non-HDLC, and triglyceride (TGL) levels among Uruguayan adolescents and assessed the role of body mass index (BMI) and sex as potential effect modifiers. Adolescents, 9-20-year-old (*n* = 327 in complete-case and 337 in imputed dataset) in Uruguay had blood As measured two times (T1 and T2), a year apart, during 2021–2023. Serum lipid markers were measured at T2. Blood As was averaged and log transformed. Separate linear regressions were built for each lipid marker. Analyses were stratified by sex and at 85th percentile of BMI to assess effect modification. Median (range) blood As at T1 and T2 were 0.38 (0.18, 4.01) µg/L and 0.36 (0.14, 4.81) µg/L, respectively. 8%, 25%, and 24% adolescents had borderline or high levels of TGL (> 150 mg/dL), TC (> 170 mg/dL), and non-HDLC (> 120 mg/dL), respectively, and 31% had low levels of HDL-C (< 45 mg/dL). Blood As showed a positive association with TGL [β (95% CI): 12.2 (0.88, 23.5)], but not other lipids. Blood As was positively associated with TGL [β (95% CI): 30.3 (8.24, 52.4)] among those with BMI≥85th percentile and with TC [12.4 (2.13, 22.6)], non-HDLC [12.0 (2.81, 21.1)] and TGL [20.3 (5.17, 35.4)] among girls. Similar findings were seen in the complete case and imputed datasets. Our study showed that low-level blood As was associated with higher TGL levels among Uruguayan adolescents with evidence of an interaction with BMI and sex.

**Supplementary Information:**

The online version contains supplementary material available at 10.1007/s12403-026-00768-x.

## Introduction

Arsenic (As) is a naturally occurring, ubiquitous metalloid to which millions of people are exposed globally. Several population-based studies have shown an association between As exposure and cardiovascular disease (CVD) incidence (Chen et al. 2013; James et al. 2015), prevalence (Tseng et al. 2003; Gong and O’Bryant 2012), and mortality (Chen et al. 1996, 2011; Nigra et al. 2021). Importantly, these associations have been found in geographic areas considered as having high- (Chen et al. 1996, 2011, 2013; Tseng et al. 2003) and low-level (Gong and O’Bryant 2012; James et al. 2015; Nigra et al. 2021) arsenic exposure. Despite this evidence, the link of As exposure measured in either in urine or blood with precursors of CVD including lipid markers is understudied and the existing studies reveal inconsistent results. For example, no difference in the levels of triglycerides (TGL), total cholesterol (TC), low-density lipoprotein (LDL-C), and high-density lipoprotein cholesterol (HDL-C) was observed between Italian workers occupationally exposed to As compared to unexposed workers (Ledda et al. 2018). Similarly, no association was found between urinary As concentrations and TGL among 18–65-year-old women from the Andean plateau in northern Argentina (Ameer et al. 2015). On the other hand, Bangladeshi adults from As-endemic areas had higher levels of oxidized LDL compared to those from non-endemic areas (Karim et al. 2013). Similarly, among U.S. adults participating in the National Health and Nutrition Examination Survey (NHANES), higher levels of urinary As were associated with higher levels of TC and LDL-C (Qu and Huang 2022). Among elderly participants in a study in China, increasing tertiles of blood As concentrations were associated with elevated levels of TC and LDL-C (Huang et al. 2024). A systematic review and meta-analysis of five studies on As exposure and lipid metabolism among adults indicated that As exposure is associated inversely with serum HDL-C and positively with serum LDL-C levels (Zhao et al. 2021).

Understanding the relationship between As exposure and lipid markers in children and adolescents is important because markers such as TC (Fuentes et al. 2003; Harrabi et al. 2010; Osawa et al. 2022), LDL-C (Harrabi et al. 2010), and TGL (Harrabi et al. 2010) track over time. Nevertheless, current evidence for the As-lipid relationship in these age groups is scarce and inconsistent. For instance, in-utero exposure to As was associated with reduced TC and HDL-C levels among 4.5 and 9-year-old Bangladeshi children in a longitudinal study (Akhtar et al. 2021), whereas 14-year-old Taiwanese adolescents in a high As exposure trajectory were at a higher risk of high levels of TC and LDL-C compared to those in a low exposure trajectory (Kuo et al. 2018). Among 12–17-year-olds participating in the 2009–2016 NHANES cycles, unmethylated inorganic As concentration in urine was positively associated with HDL-C and TC levels (Yue et al. 2022). Further understanding how As exposure is associated with lipid markers, which in turn are associated with CVD outcomes, is critical in children and adolescents. Importantly, except for the NHANES-based study, all other studies have been conducted in geographic areas that are considered to have high-level As exposure.

There is reason to suspect that overweight or obesity may act synergistically with As exposure to increase the risk of poor outcomes including unhealthy lipid profiles or CVD (Eick and Steinmaus 2020). Both As exposure and obesity have been associated with oxidative stress, inflammation, insulin resistance, and adipokine expression, which are linked with several chronic diseases (Eick and Steinmaus 2020). Studies in animal models have shown that As toxicity was exacerbated by increased body weight (Eick and Steinmaus 2020). Among humans, higher risk of As-related outcomes such as type-2 diabetes (Castriota et al. 2018), lung cancer (Steinmaus et al. 2015), bladder cancer (Steinmaus et al. 2015; Koutros et al. 2018), non-malignant respiratory symptoms such as cough, wheezing, shortness of breath (Nardone et al. 2017), as well as higher levels of the inflammatory marker soluble vascular cell adhesion molecule-1 (VCAM-1) (Wu et al. 2012) were observed among individuals with a higher Body Mass Index (BMI). Existing studies among children and adolescents do not clarify whether BMI modifies the relationship between As exposure and lipid markers.

Studies have indicated that women are more efficient in methylating (detoxifying) As from the body compared to men (Gamble et al. 2005; Steinmaus et al. 2005; Huang et al. 2008; Lindberg et al. 2008a, b; Lindberg et al. 2008a, b). Yet, differences in the association between As exposure and CVD outcomes by sex are not consistently seen in population studies. For example, no substantial difference in As-induced CVD mortality was seen between men and women in Taiwan (Cheng et al. 2010) or Spain (Medrano et al. 2010). In contrast, the association between urinary As levels and CVD incidence was stronger in women compared to men participating in the Strong Heart Study in the U.S. (Moon et al. 2013). Further, effect modification of the association between As exposure and lipids is largely unassessed. Only one study among U.S. adults (Qu and Huang 2022) and one in children and adolescents from Bangladesh (Akhtar et al. 2021) included assessment of effect modification of the association between urinary As levels and lipids by sex; neither observed differences between males and females.

The objective of this study was to examine the association between blood As levels and the serum levels of TC, HDL-C, non-HDLC, and TGL among Uruguayan adolescents, a group characterized by low-level As exposure. We also explored the role of BMI and sex as potential effect modifiers of the association between As exposure and lipid markers. We hypothesized that higher As exposure would be associated with higher serum TC, non-HDLC, and TGL, but lower HDL-C. We further hypothesized that these relationships would be exacerbated among adolescents with higher BMI and would differ between boys and girls.

## Methods

### Study Design and Participant Recruitment

The present study is based on the cohort Salud Ambiental Montevideo (SAM) who were recruited as schoolchildren from urban areas of Montevideo, Uruguay. The cohort included 682 children aged 6–8 years recruited between 2011 and 2019. These children came from low-income households and were residents of areas considered to be at risk of metal exposure. Details of cohort recruitment are provided elsewhere (Kordas et al. 2016; Desai et al. 2018).

In 2021 the SAM study participants, now aged 10–18 years, were invited for further follow-up visits where sociodemographic, anthropometric, and biochemical data were collected. This follow-up study was called the “Complex Mixtures, Oxidative Stress, and Cognition” (MOX). The first of these follow-up visits occurred in 2021–2022 and the second in 2022–2023. Of the 682 cohort participants, 421 returned for follow-up visit 1 and 399 for visit 2. Altogether, 430 unique participants joined the MOX study between 2021 and 2023. Written consent to participation and data collection was provided by those over 18 years of age. Caregivers provided consent for those younger than 18 years. The MOX study was approved by the institutional review board at the University at Buffalo (STUDY00004845) and the ethics committee at the Catholic University of Uruguay (#201230).

### Data Collection

#### Socioeconomic Status

Household socioeconomic status was assessed using a questionnaire that caregivers and participants (where applicable) filled out during their visit to the study center at the Catholic University of Uruguay. Questions pertained to ownership of 19 specific assets such as television, computer, automobile etc. An assets score was calculated using principal component analysis. Higher scores reflected a higher number of possessions. The mean (SD) score was 0.0 (1.0). Details of the scores are provided elsewhere (Rodríguez et al. 2025).

#### Body Mass Index

A portable stadiometer (Seca 214, Shorr Productions, Colombia, MD) was used to measure participants’ height in triplicate to the nearest 0.1 cm. A digital scale (Toledo model 2096, Negri, Quartino & Ferrari, Montevideo, Uruguay) was used to measure their weight in triplicate to the nearest 0.1 kg. Both measurements were carried out by a nutritionist at the study center. The adjusted weight was calculated by subtracting the standard weights for the clothing worn by participants during the visit from the average of the three weight measures. The average of the three height measures was also calculated. BMI was calculated using the average height and weight measures.

#### Secondhand Smoke Exposure

Exposure to secondhand smoke was estimated using a questionnaire administered to participants when they visited the study center. The questions pertained to smoking behaviors inside vs. outside the house, the number of smokers in the household, the type of smoking device used (cigarettes, e-cigarettes, cigars), the number of people who smoked indoors, and the frequency of indoor smoking. To estimate secondhand smoke exposure, the number of smokers indoors was counted and the amount of time that the participant was exposed to smoke indoors was evaluated for a typical weekday as well as weekend day. The exposure time was estimated based on the following categories: never; <1 h/day; 1–2 h/day; 3–5 h/day; 5 + hours/day. Numerical values were assigned to the responses to the question about exposure time and the values were summed to range between 0 and 8. The overall secondhand smoke exposure was categorized based on the responses as none, low (1–2 points), and high (3 + points). Secondhand smoke exposure inside the home was assumed to be from cigarettes because only one father and no mothers reported using e-cigarettes, and no parents reported using cigars in the entire cohort.

#### Physical Activity

Physical activity levels were assessed using the Physical Activity Questionnaire for older Children (PAQ-C) (Kowalski et al. 2004) adapted for the Uruguayan population. The questionnaire consisted of five questions, and the scoring system was based on the procedures for the PAQ-C. Question 1 pertained to taking part in specific sports such as swimming, soccer, basketball etc. Sports that were specific to the U.S. and/or uncommon in Uruguay, such as ice hockey, cross-country skiing, floor hockey, street hockey, American football, baseball, tag, rowing, and badminton were removed whereas horse riding was added. The mean score for activities in question 1 was calculated based on participants’ responses. Questions 2–5 inquired about participation in sports at specific times and settings such as after school, on weekends etc. The final physical activity scores were calculated by averaging the scores from all questions.

#### Blood Arsenic and Blood Lead (Pb) Concentrations

Phlebotomists collected fasting venous blood samples from participants during the two follow up visits which occurred approximately a year apart at the study center. Lavender-top tubes containing an ethylenediaminetetraacetic acid anticoagulant were used to store the collected blood (approximately 2 ml). These tubes were pre-screened by the laboratory to detect low-level trace elements for this study. The blood samples were mixed with the anticoagulant by inverting the tubes 4–6 times and then refrigerated at 4 °C. Blood samples were shipped to the Trace Elements Section of the Laboratory of Inorganic and Nuclear Chemistry at the New York State Department of Health’s Wadsworth Center every month. Once received, the samples were transferred to pre-screened polypropylene tubes and stored at −80 °C until further processing and analysis.

Whole blood specimens were shipped to the Laboratory of Inorganic and Nuclear Chemistry at the Wadsworth Center, New York State Department of Health (Albany, NY). Specimens were prepared for analysis in a SterilGARD^®^ e3 Class II, Type A2 Biological Safety Cabinet (The Baker Company, Sanford, MA) which meets ISO 5 (Class 100) standards. All other preparation work was performed under Class 100 clean room conditions or better (Terra Universal, Fullerton, CA). Trace element analysis was carried out on a Thermo Scientific iCAP™ TQ inductively coupled plasma – tandem mass spectrometer (ICP-MS/MS), using an analytical method validated for 23 trace elements including lead (Pb) and arsenic (As), and optimized for human biomonitoring studies. Multielement calibration standards were prepared from a NIST-traceable (i.e., National Institute of Standards and Technology) stock solution (High Purity Standards, Charleston, SC, USA) and an eight-point, base-blood matrix-matched calibration curve used for each element (1 + 49 dilution of blood sample to diluent). For blood Pb measurements, the sum of three major stable isotope (^206^Pb^+^+^207^Pb^+^+^208^Pb^+^) was monitored in KED gas mode with ^193^Ir^+^ as the internal standard. For blood As, which is monoisotopic, the measurements were made using the TQ-O2 gas mode while monitoring the product (^75^As^16^O^+^) at m/z 91, i.e., mass shift mode. Full details of the ICP-MS/MS parameters for Pb and As are given in Supplemental Tables 1, 2, and 3.

Tri-level blood-based quality control (QC) materials were analyzed at the beginning, mid-point and end of each analytical run, to ensure that results were obtained under conditions of repeatability. All elevated results were confirmed by reanalysis of new aliquot per laboratory policy. A random number (2%) of samples were analyzed in duplicate to monitor repeatability. Archived proficiency testing (PT) samples were also analyzed to monitor performance. Method accuracy was assessed using NIST SRM 955c (Toxic Metals in Frozen Caprine Blood), and NIST 955 d (Toxic Elements and Metabolites in Frozen Human Blood.

The Wadsworth laboratory participates successfully in numerous PT programs and external quality assessment (EQA) schemes for trace elements in blood including those operated by: the New York State Department of Health, Albany, NY (NYSDOH); L’Institut National de Santé Publique du Québec, Le Centre de Toxicologie du Québec (CTQ); the Centers for Disease Control and Prevention, Lead, Cadmium, Manganese and Mercury PT program (LAMP); the Trace Elements External Quality Assessment Scheme, at the University of Surrey, UK; and the German External Quality Assessment Scheme, operated by the Institute and Outpatient Clinic for Occupational, Social and Environmental Medicine of the Friedrich-Alexander University, Erlangen-Nuremberg, Germany.

#### Serum Lipid Markers

##### Serum Collection

Lipid markers were measured using blood samples collected during visit 2. Fasting venous blood was collected in a tube that contained a coagulant and separator gel. The tube was left to stand for approximately 45 min at room temperature to facilitate coagulation of blood. Then the tube was spun at 3000 rpm to separate serum from the clot. Serum samples were aliquoted and stored at −80 °C. Samples were shipped on dry ice to the CERLab, Department of Laboratory Medicine, Boston Children’s Hospital for analysis, and analyzed on the Roche Cobas 6000 system using reagents and calibrators from Roche Diagnostics (Indianapolis, IN). The laboratory methods are briefly outlined below, with additional details provided in Online Supplemental Material.

##### Serum Total Cholesterol

Total cholesterol was measured enzymatically; the specificity of the enzymatic reaction was combined with peroxidase/phenol-4-aminophenazone indicator reaction. Free cholesterol was produced when cholesterol esters were hydrolyzed by cholesterol esterase. Cholesterol oxidized to cholest-4-en-3-one and hydrogen peroxide in the presence of oxygen and cholesterol oxidase. Hydrogen peroxide then reacted with a dye to produce a quinoneimine dye; the intensity of its color was measured at 505 nm. It was directly proportional to the concentration of cholesterol in the sample.

##### Serum Triglycerides

Triglycerides were measured enzymatically and correction for endogenous glycerol was carried out. In the presence of glycerol kinase and adenosine triphosphate, the endogenous glycerol was phosphorylated to produce glycerol-3-phosphate in a preliminary reaction. It then oxidized to generate hydrogen peroxide, which reacted with 4-chlorophenol to produce an oxidative product. Then, lipase mixture hydrolyzed triglycerides to produce glycerol and fatty acids in the actual assay reaction. Similar to the preliminary reaction, glycerol kinase phosphorylated glycerol to produce glycerol-3-phosphate. It then oxidized to generate hydrogen peroxide. A colored product was generated when hydrogen peroxide reacted with a dye. The intensity of the generated color was measured at 505 nm. It was directly proportional to the concentration of triglycerides in the sample.

##### Serum High Density Lipoprotein Cholesterol

High Density Lipoprotein Cholesterol concentrations were measured using a direct enzymatic colorimetric assay. In this technique, soluble complexes of non-HDL lipoproteins [low-density lipoproteins (LDL), very low-density lipoproteins (VLDL) and chylomicrons] and sulfated alpha-cyclodextrin-Mg + + were formed. Polyethylene glycol (PEG)-modified cholesterol oxidase and esterase were used to determine the cholesterol component of HDL. Both PEG-modified cholesterol oxidase and esterase had limited reactivity with the complexed apolipoprotein B-containing lipoproteins.

##### Non-High Density Lipoprotein Cholesterol

Non-High Density Lipoprotein Cholesterol was calculated as (Total Cholesterol minus High Density Lipoprotein Cholesterol) (Virani 2011). It is regarded as a measure of the cholesterol content of all atherogenic lipoproteins and is shown to be a reliable marker of coronary artery disease risk (Virani 2011).

### Statistical Analysis

#### Sample Size

**Fig. 1:**
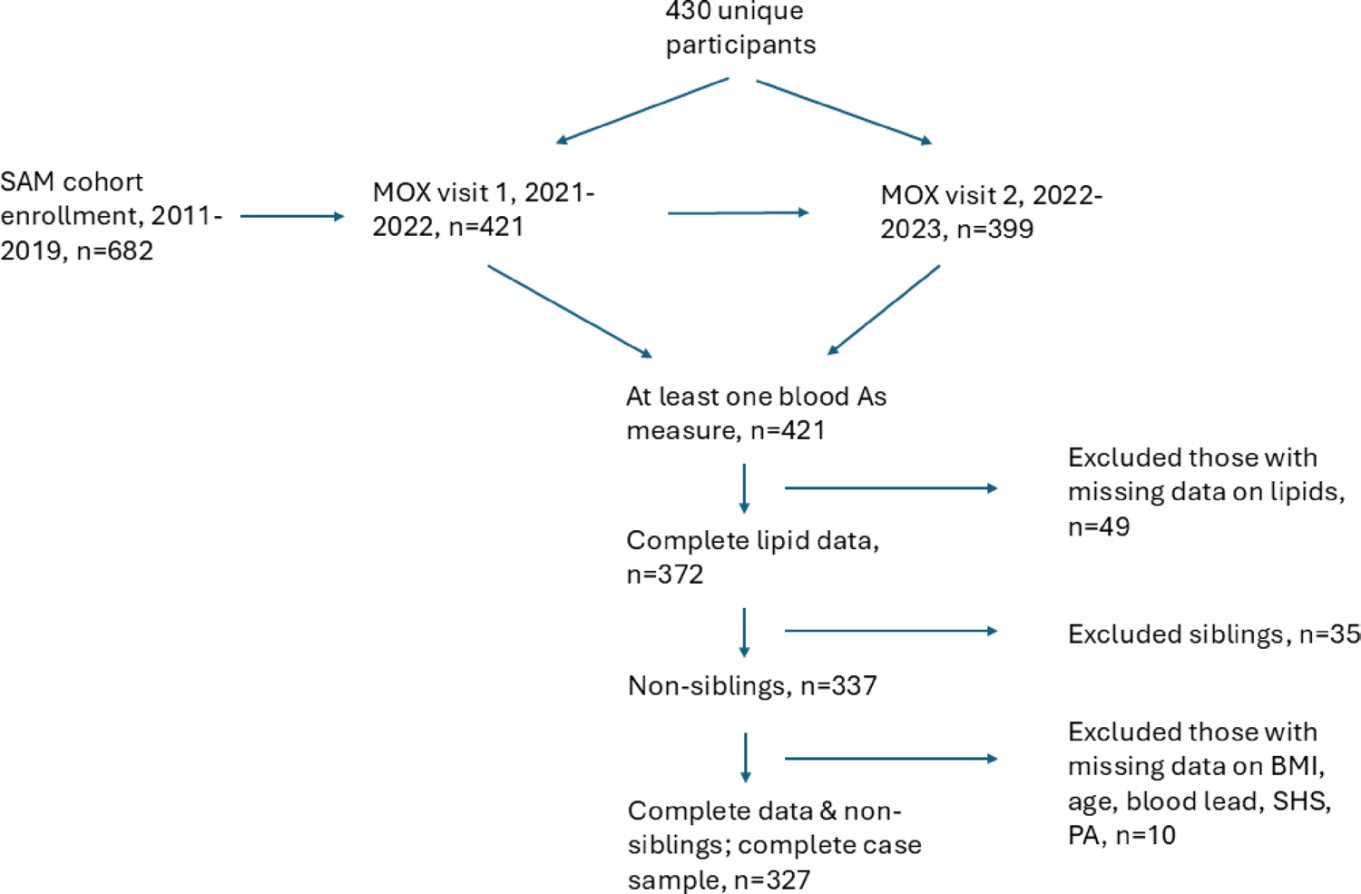
Overview of the study timeline and the process of reaching the complete case sample. BMI: Body mass index; SHS: Secondhand smoke; PA: Physical activity

Figure 1 presents the process of arriving at the complete case sample of 327 participants. In short, 421 participants had a blood As measure at either visit (404 at visit 1; 378 at visit 2; and 319 at both visits). Of the 421 participants with any measure of As, 49 with missing data on lipid markers were excluded. The sample size was further reduced to 337 upon excluding 35 siblings of existing participants to maintain the assumption of independent observations in regression models. Ten participants with missing data on covariates such as BMI at visit 2, age, blood Pb levels measured at either visit, secondhand smoke exposure and physical activity score were further excluded, leading to a complete case sample of 327. Data were imputed for the 10 participants with missing observations on covariates using multiple imputations with chained equations in STATA 18, leading to a sample size of 337 participants in the imputed dataset.

#### Analyses

Analyses in the complete case dataset were carried out using SAS version 9.4 (SAS Institute Inc., Cary, NC, USA) whereas those in the imputed dataset were carried out using STATA 18. Descriptive analyses included calculating medians (range) for continuous variables, and frequencies (percentage) for categorical variables in the complete case dataset. Pearson’s correlations were carried out to describe the interrelationships among lipid markers. Blood As concentrations from the two time points were averaged and log-transformed for analysis. Separate linear regression models were built for TC, HDL-C, non-HDLC, and TGL, adjusting for sex (boys/girls), age (continuous), BMI at visit 2 (continuous), secondhand smoke exposure (none/low/high), physical activity score (continuous), household assets score (continuous), and blood Pb levels (continuous) in the complete case and imputed datasets. These covariates were selected based on existing literature (Kuo et al. 2018; Akhtar et al. 2021; Yue et al. 2022).

Effect modification by BMI was evaluated by conducting stratified analyses; As-lipid associations were tested separately among those with BMI < 85th and BMI ≥ 85th percentile. BMI ≥ 85th percentile but < 95th percentile is defined as overweight and BMI > 95th percentile is defined as obese according to age-appropriate definitions of overweight and obesity (CDC 2024). Effect modification by sex was evaluated also by conducting stratified analyses; As-lipid associations were tested separately among boys and girls.

Lastly, as sensitivity analysis, regression models assessing the associations between averaged, log-transformed blood As levels and lipid markers were repeated among participants that had blood As measured at both visits (*n* = 319).

## Results

**Fig. 2:**
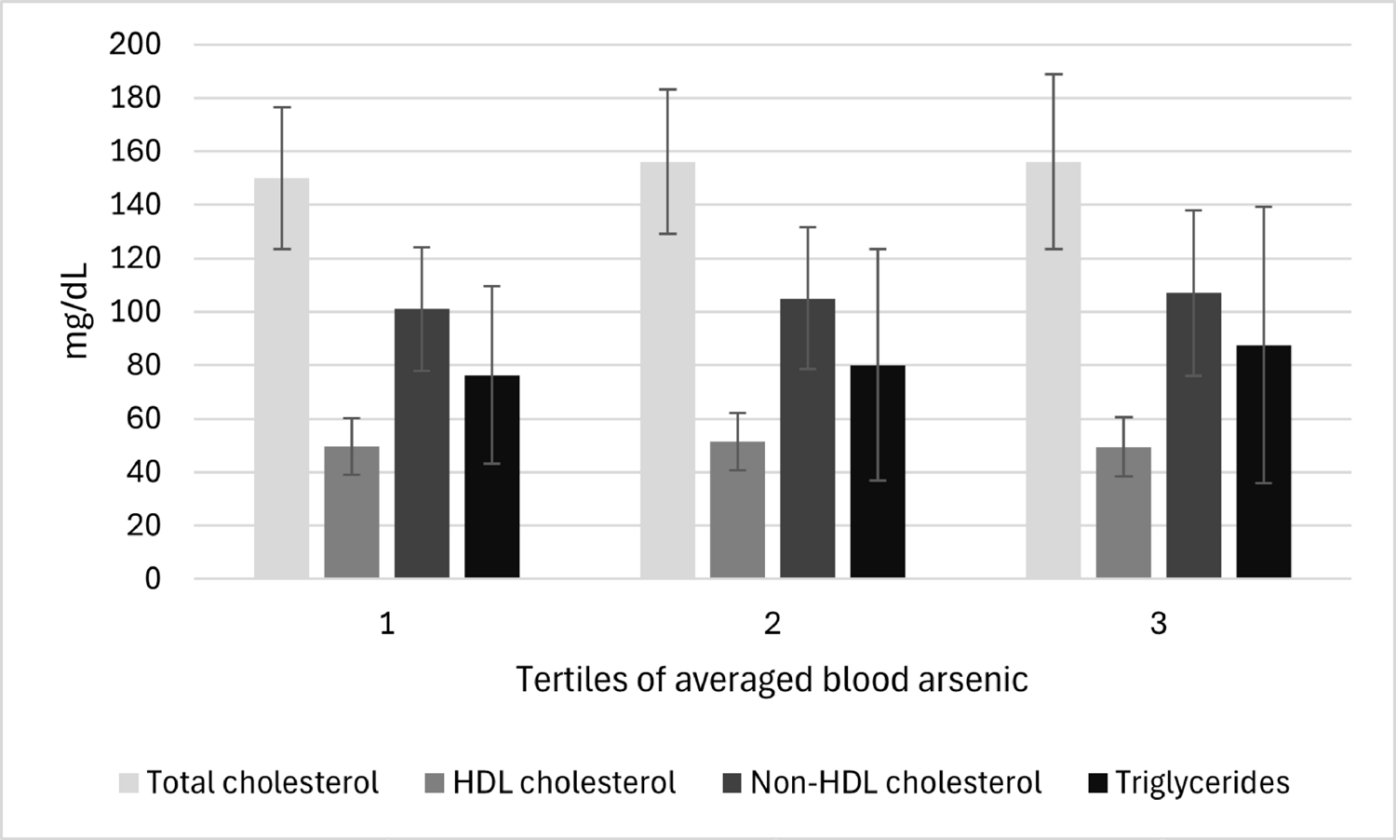
Mean (SD) lipid marker levels according to tertiles of averaged blood As levels among Uruguayan adolescents (*n* = 327)

Sociodemographic, biochemical, and anthropometric characteristics of participants in the complete case sample (*n* = 327) are presented in Table 1. The median (range) age was 11.7 (8.59, 19.8) years with an equal distribution of boys (49.5%) and girls (50.5%). Median (range) blood As levels were 0.38 µg/L (0.18, 4.01) and 0.36 µg/L (0.14, 4.81) whereas blood Pb levels were 1.17 µg/dL (0.28, 43.2) and 1.03 µg/dL (0.39, 24.8) at visit 1 and 2, respectively. Median (range) serum TC, HDL-C, non-HDLC, and TGL levels were 153 (81.0, 302) mg/dL, 49.0 (21.0, 93.0) mg/dL, 101 (42.0, 260) mg/dL, and 70.0 (21.0, 291) mg/dL, respectively. Based on age-appropriate guidelines, 8%, 25%, and 24% adolescents had borderline or high levels of TGL (> 150 mg/dL), TC (> 170 mg/dL), and non-HDLC (> 120 mg/dL) respectively, and 31% had low levels of HDL-C (< 45 mg/dL). TC levels showed a moderate positive correlation with HDL-C (*r* = 0.35, *p* < 0.001) and TGL (*r* = 0.33, *p* < 0.001) and a strong positive correlation with non-HDLC (*r* = 0.93, *p* < 0.001). TGL levels were inversely correlated with HDL-C (*r*=−0.37, *p* < 0.001) and positively correlated with non-HDLC (*r* = 0.50, *p* < 0.001), similar to the correlations seen in the literature (Amusat 2021). Blood As levels and levels of serum lipid markers by participant characteristics are presented in Supplemental Table 4. Notably, triglyceride levels differed between boys and girls [median (range): 65.0 (21.0, 291) and 74.0 (25.0, 261), respectively], and among those with BMI < 20.3 kg/m2 and those with BMI ≥ 20.3 kg/m2 [median (range): 64.0 (21.0, 181) and 75.0 (30.0, 291), respectively]. The other parameters remained similar across participants by various characteristics. Supplemental Table 5 presents the comparison of sociodemographic, anthropometric, and biochemical characteristics of participants included in the complete case sample and those excluded from the complete case sample; participant characteristics were similar in both groups. Figure 2 presents the mean (SD) lipid marker levels according to tertiles of averaged blood As levels (not log-transformed), the detailed summary statistics are also presented in Supplemental Table 6.

**Table 1:** Characteristics of Uruguayan study participants in the complete case sample (*n* = 327)^*^

Variables	*N*	Value median (range) or *n* (%)
Age, years	327	11.7 (8.59, 19.8)
Sex		
Girls Boys	165 162	165 (50.5%) 162 (49.5%)
Blood As at time 1, µg/L	319	0.38 (0.18, 4.01)
Blood As at time 2, µg/L	327	0.36 (0.14, 4.81)
Blood Pb at time 1, µg/dL	319	1.17 (0.28, 43.2)
Blood Pb at time 2, µg/dL	327	1.03 (0.39, 24.8)
BMI, kg/m^2^	327	20.3 (13.0, 43.5)
Secondhand smoke exposure		
None Low High	129 129 69	129 (39.5%) 129 (39.5%) 69.0 (21.1%)
Physical Activity Score	327	1.64 (0.94, 2.59)
Household Assets Score	327	0.18 (−2.26, 1.69)
Total cholesterol, mg/dL	327	153 (81.0, 302)
HDL cholesterol, mg/dL	327	49.0 (21.0, 93.0)
Non-HDL cholesterol, mg/dL	327	101 (42.0, 260)
Triglycerides, mg/dL	327	70.0 (21.0, 291)

^*^The complete case sample is defined as participants with at least one blood arsenic measure available. At visit 1, blood levels of arsenic and lead were available for 319 of 327 participants in this sample.

Abbreviations in the order they appear in the table: *As* arsenic; *Pb* lead; *BMI* body mass index; *HDL* high-density lipoprotein

The association between averaged, log-transformed blood As concentrations and lipids was assessed in both the complete case (*n* = 327) and the imputed (*n* = 337) dataset; similar findings were obtained in both as shown in Table 2. No association between As exposure and HDL-C was observed. There was a positive association between blood As levels and TGL in the complete case sample [β (95% CI): 12.2 (0.88, 23.5)] after adjusting for covariates. A similar albeit non-statistically significant association was observed in the imputed sample [β (95% CI): 10.3 (−0.67, 21.2)]. The covariate-adjusted associations of As exposure with TC [β (95% CI): 6.77 (−1.15, 14.7)] and non-HDLC [β (95% CI): 6.65 (−0.69, 14.0)] were positive and borderline statistically significant in the complete case dataset. The direction of the association remained the same in the imputed dataset; however, they did not reach statistical significance. Confidence intervals indicated a level of imprecision in the estimates.

**Table 2:** Association between averaged, log-transformed blood arsenic concentrations and lipids among Uruguayan study participants

	Complete case dataset (*n* = 327)		Imputed dataset (*n* = 337)	
	Crude β (95% CI)	Adjusted^1^ β (95% CI)	Crude β (95% CI)	Adjusted^1^ β (95% CI)
Total cholesterol	7.05 (−0.75, 14.9)^#^	6.77 (−1.15, 14.7)^#^	5.81 (−1.75, 13.4)	5.63 (−2.04, 13.3)
HDL cholesterol	−0.37 (−3.32, 2.57)	0.11 (−2.77, 3.00)	−0.68 (−3.56, 2.20)	−0.07 (−2.89, 2.75)
Non-HDL cholesterol	7.43 (0.13, 14.7)^*^	6.65 (−0.69, 14.0)^#^	6.48 (−0.55, 13.5)	5.70 (−1.39, 12.8)
Triglycerides	13.5 (1.84, 25.2)^*^	12.2 (0.88, 23.5)^*^	11.9 (0.62, 23.1)^*^	10.3 (−0.67, 21.2)^#^

*HDL* high-density lipoprotein

^1^Adjusted for sex, age, body mass index, secondhand smoke exposure, physical activity score, household assets score, and average blood lead levels from the two measures (one measure if two were unavailable)

^*^p < 0.05

^*#*^*p* < 0.1

**Fig. 3:**
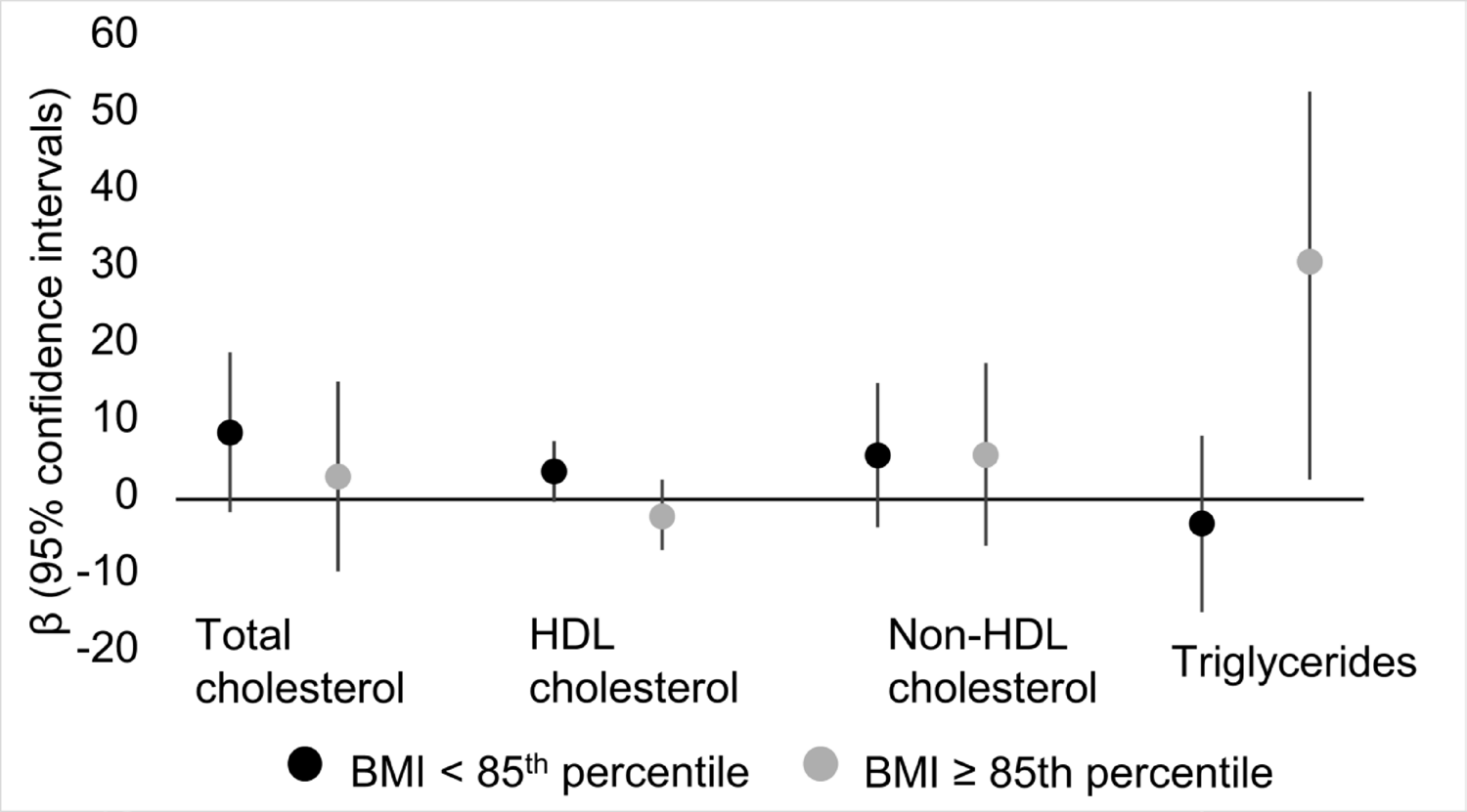
Associations^1^ between averaged, log-transformed blood arsenic concentrations and lipids stratified by body mass index among Uruguayan study participants in the complete case sample (*n* = 327). BMI: body mass index; HDL: high-density lipoprotein. ^1^Adjusted for sex, age, secondhand smoke exposure, physical activity score, household assets score, and average blood lead levels from the two measures (one measure if two were unavailable)

Results of the regression analyses stratified based on BMI (< 85th percentile vs. ≥85th percentile) are presented in Supplemental Table 7. Positive associations were seen between As exposure and TGL levels among adolescents with BMI ≥ 85th percentile in the complete case [β (95% CI): 30.3 (8.24, 52.4)] and imputed datasets [β (95% CI): 23.9 (3.17, 44.7)] upon adjusting for covariates. Conversely, among adolescents with BMI< 85th percentile, an inverse and non-statistically significant association was seen between As exposure and TGL levels in the complete case [β (95% CI): −3.79 (−15.3, 7.72)] and imputed datasets [β (95% CI): −3.20 (−14.5, 8.14)] after adjusting for covariates. The confidence intervals for TGL showed little overlap between the strata, suggesting the presence of effect modification. Results of the BMI-stratified analysis in the complete case sample (*n* = 327) are presented in Fig. 3.

**Fig. 4:**
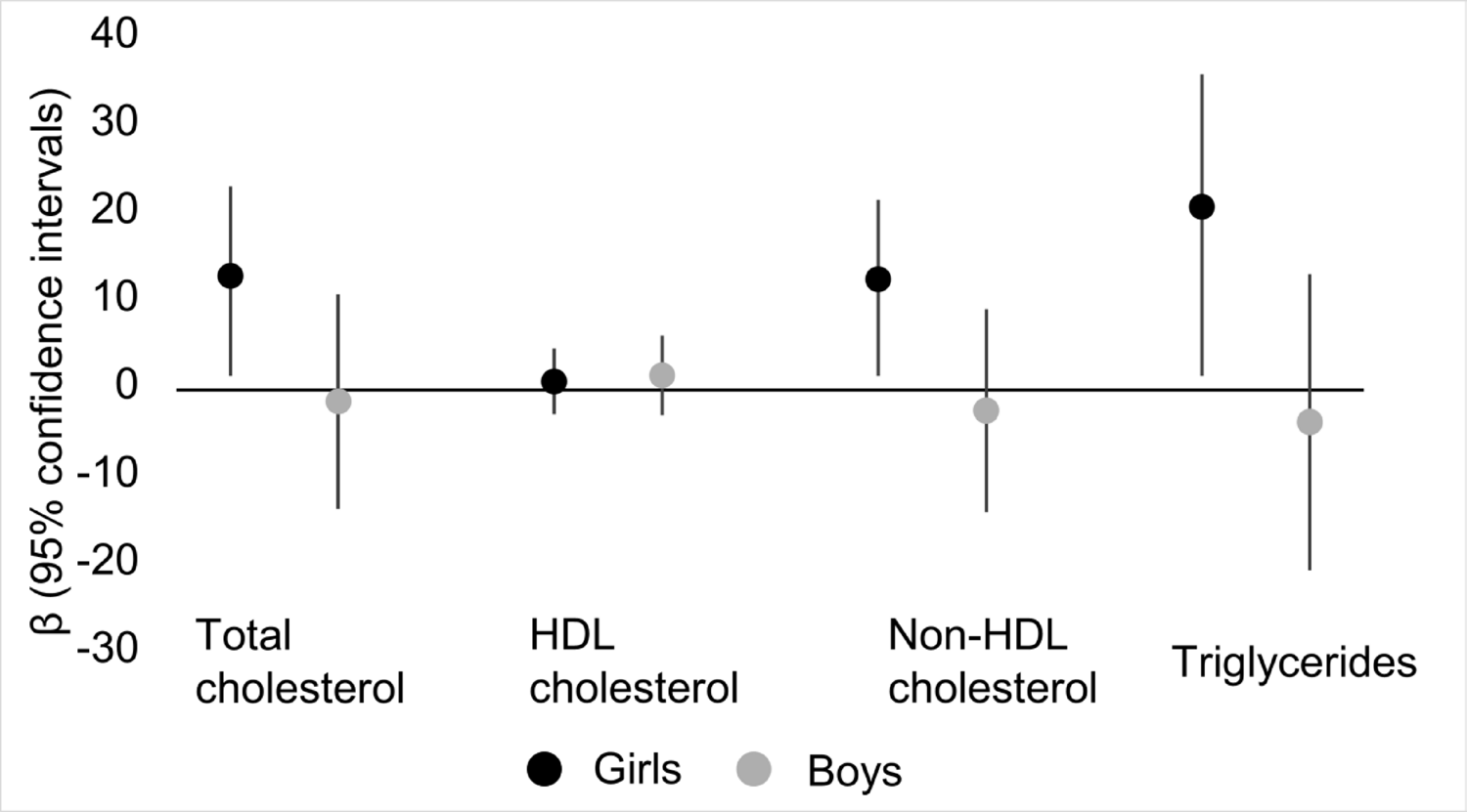
Associations^1^ between averaged, log-transformed blood arsenic concentrations and lipids stratified by sex among Uruguayan study participants in the complete case sample (*n* = 327). HDL: high-density lipoprotein. ^1^Adjusted for age, body mass index, secondhand smoke exposure, physical activity score, household assets score, and average blood lead levels from the two measures (one measure if two were unavailable)

Supplemental Table 8 presents the As-lipid associations stratified by sex in the complete case and imputed datasets. Among girls, As exposure was positively associated with levels of TC [[β (95% CI): 12.4 (2.13, 22.6)], non-HDL cholesterol [β (95% CI): 12.0 (2.81, 21.1)] and TGL [β (95% CI): 20.3 (5.17, 35.4)] upon adjusting for covariates in the complete case dataset. Conversely, inverse albeit non-statistically significant associations of As exposure were seen with levels of TC [[β (95% CI): −1.89 (−14.1, 10.3)], non-HDL cholesterol [β (95% CI): −2.95 (−14.5, 8.63)] and TGL [β (95% CI): −4.25 (−21.1, 12.6)] in covariate-adjusted models among boys. The confidence intervals for TC, non-HDL cholesterol and TGL showed some degree of non-overlap, suggesting the presence of effect modification. Model estimates in the imputed dataset were consistent with these findings. Results of the sex-stratified analysis in the complete case sample (*n* = 327) are presented in Fig. 4.

Supplemental Table 9 presents the associations between averaged, log-transformed blood As concentrations and lipids among participants with As measures available at both visits (*n* = 319), which are consistent with findings in the complete case sample.

## Discussion

In a group of Uruguayan adolescents characterized by low-level As exposure, blood As levels were positively associated with serum TGL levels. There was an indication of a positive association between As exposure and TC and non-HDLC levels; these associations were borderline statistically significant. No associations were observed between As exposure and HDL-C levels. Notably, blood As showed a positive association with TGL levels among those with BMI ≥ 85th percentile and with TC, non-HDLC, and TGL levels among girls. The magnitude of these associations should be interpreted with caution, however, given the wide confidence intervals likely resulting from the modest sample size.

Ours is one of the few studies on the link between As exposure and serum lipids conducted among adolescents (Kuo et al. 2018; Akhtar et al. 2021; Yue et al. 2022). Maternal urinary As concentrations during pregnancy as well as childhood urinary As concentrations at ages 4.5 years and 9 years showed inverse associations with HDL-C levels measured at 9 years of age among Bangladeshi children exposed to high levels of As (median urinary As levels during the prenatal period were 76.07 µg/L; at 4.5 years, 57.05 µg/L; at 9 years, 52.91 µg/L) (Akhtar et al. 2021). Our findings do not align with these; we found no associations between blood As levels and HDL-C levels. In addition to age, a major difference between the study in Bangladesh and our study is the very high levels of As exposure compared to participants in Uruguay. When SAM children were ~ 7 years of age, median (IQR) urinary As levels were 11.9 (9.8) µg/L (Desai et al. 2018), thus 5–6 times lower than in Bangladesh.

In a study in Taiwan, adolescents were categorized into exposure trajectories using their urinary As concentrations collected at multiple points during a 15-year period (Kuo et al. 2018). The trajectories were calculated based on the likelihood of having total urinary As levels higher than the population median at each follow-up visit (Kuo et al. 2018). Those on a rising-high As exposure trajectory, i.e. participants whose probability of having As exposure higher than the median increased with time, showed a positive association with TC compared to those on a stable-low exposure trajectory, i.e., those with consistently low likelihood of exceeding the median As level (Kuo et al. 2018). Again, our participants had low-level As exposure, shorter period of follow-up, and a distinct exposure biomarker, thus precluding direct comparisons with the study in Taiwan. Finally, among 12–17-year-olds participating in the 2009–2016 NHANES cycles, a positive association between unmethylated inorganic As exposure and TC and HDL-C levels was observed (Yue et al. 2022). For the overall sample, our findings are not consistent with the NHANES study despite having more comparable exposure [median (95% CI) urinary As in NHANES participants was 5.18 µg/L (4.81, 5.56) vs. median (IQR) urinary As in our original SAM cohort at age ~ 7 years of 11.9 (9.8) µg/L]. During adolescence, our study participants had very low levels of blood As [median (range) at visit 2: 0.36 µg/L (0.14, 4.81)]. Despite the wide 95% confidence intervals, our findings indicate an association between low-level As exposure and TC among girls. Among NHANES participants, As exposure was measured from a spot urine sample, whereas in our study, blood As concentrations (i.e., total As) were averaged over two points approximately a year apart. Urinary As speciation analysis enables snapshots into the As methylation process and therefore the NHANES-based study controlled for measures of dimethylarsinic acid. Because our study relied on total As in blood, our models did not account for the products in the As methylation cycle. These differences between the studies could explain the inconsistent findings to some extent.

The biological mechanism underlying the association between As exposure and lipid markers is not clearly understood. Studies in animal models have shown that chronic exposure to As led to dyslipidemia; the levels of lysophospholipids, phosphatidylcholines, and TGL as well as lipid oxidation increased in rats exposed to 3 ppm of As in drinking water during the prenatal period and early life compared to unexposed rats (Rivas-Santiago et al. 2019). Similarly, dyslipidemia was observed among rats exposed to trivalent (100 ppm) and pentavalent forms (150 ppm) of inorganic As in drinking water (Afolabi et al. 2015) and disrupted lipid metabolism was seen among rats exposed to 0.5, 2 or 10 ppm sodium arsenite, a trivalent form of inorganic As in another study (Wang et al. 2015). Notably, the effects in animal studies were observed at higher levels of exposure than those typically reported in human populations. Among humans, several mechanisms of As-induced dyslipidemia are proposed, including upsurge in the release of proinflammatory cytokines such as tumor necrosis factor alpha, interleukin-6, and interleukin-8 seen upon treatment of cells with trivalent As species for 2, 4, 6 and 24 h (Calatayud et al. 2014), in turn linked to dyslipidemia (Esteve et al. 2005). Oxidative stress-induced DNA damage in the vascular smooth muscle cells is another potential mechanism that has been observed upon 4-hour treatment with arsenite at a concentration above 1 µmol/L (Lynn et al. 2000). The association of As exposure (urinary As 5.5 µg/g creatinine among pregnant women in the Navajo Birth Cohort Study) with oxidative stress and lipid peroxidation was also observed (Dashner-Titus et al. 2018).

To our knowledge, ours is the only study on the As-lipids association to evaluate effect modification by BMI. Currently, the mechanism behind the synergistic effects of As exposure and obesity on various outcomes is not clear (Eick and Steinmaus 2020). Yet, several studies in animal models have shown this synergistic action (Paul et al. 2011; Tan et al. 2011; Huang et al. 2018; Xenakis et al. 2022; Calderón-DuPont et al. 2023). Among humans, it is hypothesized that As exposure and obesity have common mechanisms of action (Eick and Steinmaus 2020). For example, like As, obesity is associated with increased levels of pro-inflammatory markers and decreased mitochondrial function (Castriota et al. 2018). We observed effect modification of the association between As exposure and TGL levels by BMI, where blood As was positively associated with TGL levels among adolescents with BMI ≥ 85th percentile. The confidence intervals associated with the regression coefficients were wide, indicating a level of imprecision. Further confirmatory studies are needed, as is additional work to elucidate the synergistic action of low-level As exposure and obesity.

Differences in the As-lipid associations by sex have largely not been evaluated in either adults (Karim et al. 2013; Ameer et al. 2015; Ledda et al. 2018) or adolescents (Kuo et al. 2018; Yue et al. 2022). Only one study among adults participating in the 2003–2020 NHANES cycles (Qu and Huang 2022) and one in children and adolescents from Bangladesh (Akhtar et al. 2021) assessed sex differences in the association between urinary As levels and lipids; none were observed. We found a positive association of As exposure with TC, non-HDLC, and TG levels among girls, but null associations among boys. The confidence intervals associated with the estimates showed limited overlap, suggesting possible effect modification. Several studies have indicated that women have a better capacity to methylate, i.e., detoxify As from the body compared to men (Gamble et al. 2005; Steinmaus et al. 2005; Huang et al. 2008; Lindberg et al. 2008a, b; Lindberg et al. 2008a, b). The role of estrogen is hypothesized to underly these differences in As methylation. Estrogen stimulates endogenous choline production in the body. Endogenous choline then transforms into betaine, which helps in converting homocysteine to methionine (Fischer et al. 2007; Vahter 2007; Li et al. 2008). Methionine is a key compound in the one carbon cycle, the biochemical pathway through which the body methylates and detoxifies As, thereby enabling women to methylate As more efficiently than men (Tseng 2009). Given the pubertal age range of our participants, the role of estrogen could help explain our findings. Future studies should evaluate the role of pubertal developmental stage in the aforementioned associations. Additional studies are needed to fully elucidate these relationships.

Our study relied on the average of blood As levels measured at two visits approximately a year apart. The gold standard for measuring As exposure is in urine collected over 24 h (Johansson et al. 1998; National Research Council 1999, Ellingsen et al. 2023), although spot urine samples are generally used in large population-based studies (National Research Council 1999). While speciated As concentrations such as monomethylarsonic acid and dimethylarsinic acid can be measured in blood, the process is complex. A major concern in this process is to release the trivalent metabolites of inorganic As and monomethylarsonic acid, which are protein-bound, without altering their chemical form (Hall et al. 2006; Desai et al. 2024). Another issue is that some speciated metabolites of inorganic As, dimethylarsinic acid in particular, have a very short half-life of about an hour (Pomroy et al. 1980; Desai et al. 2024). Therefore, using blood As to reflect As methylation status is difficult. Blood As is considered a marker of recent exposure (Desai et al. 2024). Despite these limitations, there are advantages of using blood As to reflect exposure: blood As concentrations are thought to provide a more detailed and complete picture of a person’s internal dose, coming from exogenous sources as well as from some tissue compartments (National Research Council 1999, Hall et al. 2006; Desai et al. 2024). Furthermore, blood As levels reach a stable state and are a good indicator of ongoing total As exposure in populations that have a continual exposure through water and food (National Research Council 1999, Hall et al. 2006; Gardner et al. 2011; Desai et al. 2024). In our study, the correlation between blood As levels at the two visits was modest (*r* = 0.26) likely because blood As is a marker of short-term exposure and the visits were approximately one year apart; averaging the measures gave us an indication of the overall exposure during this time frame.

Our study has several strengths. It is one of the few studies evaluating the association between As exposure and lipid markers among adolescents and the only study to evaluate the role of BMI as a modifier of the association. Blood As concentrations were measured at two time points using sensitive laboratory techniques well suited for human biomonitoring of trace element exposure. Although lipid marker levels can show a within-day variation of 2–3% and are influenced by factors such as posture during the blood draw (Deeg 2006), using non-fasting lipid markers in the general population is acceptable because they are stable and vary minimally in response to regular activities, including normal food intake (Langsted et al. 2008). Our analytical models included several important covariates, including blood Pb levels and physical activity scores, which are independent risk factors for dyslipidemia (Peters et al. 2012; Kim et al. 2022; Zhang et al. 2022; Barbosa et al. 2023).

Our study also has limitations. First, we had a modest sample size of 327 participants in the complete case sample and 337 participants in the imputed dataset, likely limiting our ability to get precise estimates, particularly given that our participants had low-level exposure. Second, about 62% of participants from the original SAM cohort returned for visit 1 of the current MOX study (*n* = 421). Of these, participants with missing data on As and lipid markers were excluded, leading to a complete case sample of 327 participants. Concerns of selection bias need to be addressed if participation decisions were made based on individuals’ exposure and outcome status. However, As exposure is not routinely tested in Uruguay, and lipid markers are also not closely tracked among adolescents in this population. As a result, although not impossible, it is unlikely that participation in the study was associated with As exposure and lipid markers leading to selection bias. Third, rice (Davis et al. 2017) and seafood (Taylor et al. 2017) are known sources of As exposure. In the original SAM cohort, rice intake was associated with higher levels of total and inorganic As, as well as dimethylarsinic acid in urine (Kordas et al. 2016). In the current study sample, dietary intake data were not available, precluding our understanding of how dietary factors impacted As exposure or lipid markers. Fourth, we used total As measures in blood that did not include concentrations of speciated As such as monomethylarsonic acid and dimethylarsinic acid. Speciated As concentrations are easier to measure in urine samples than in blood. They also give an insight into the individual’s ability to metabolize and detoxify As. This lack of information reduces the comparability of our study with previous reports that relied on urinary As concentrations. Lastly, we averaged two blood As measures collected at two visits approximately a year apart and evaluated its association with lipid markers measured at visit 2. Given that blood As is a marker of recent exposure and a single measure of lipid markers in blood might not indicate an individual’s lipid profile reliably, there is a possibility that our findings did not precisely reflect the subtle biological changes in lipid makers that could have resulted from low-level As exposure. Availability of As exposure during the prenatal period and earlier in childhood, along with repeated measures of lipid markers would have enabled us to comment on causality between As exposure and serum lipid levels. Longitudinal studies in similar, low-level exposure settings with larger sample sizes and those including dietary intake data are needed to confirm our findings.

## Conclusion

At low-level exposure, blood As was associated with higher TGL levels among Uruguayan adolescents. Further, blood As showed a positive association with TGL levels among those with BMI ≥ 85th percentile and with TC, non-HDLC, and TGL levels among girls.

## Supplementary Information

Below is the link to the electronic supplementary material.

Supplementary material 1 (DOCX 50.4 kb)

## Data Availability

The datasets generated during and/or analyzed during the current study are not publicly available but are available from the corresponding author on reasonable request.
