# Supplementary material for: Association of Blood Arsenic Concentrations with Lipid Markers in Uruguayan Adolescents: Exploring Effect Modification by Body Mass Index and Sex
Source: Expo Health. 2026 Apr 14;18(2):33. doi: 10.1007/s12403-026-00768-x (PMC13079552; doi:10.1007/s12403-026-00768-x)
Supplement: Supplementary file 1 — Supplementary material 1 (DOCX 50.4 kb) [file 12403_2026_768_MOESM1_ESM.docx]

**Association of Blood Arsenic Concentrations with Lipid Markers in Uruguayan Adolescents: Exploring Effect Modification by Body Mass Index and Sex**

**Online Supplemental Material**

Gauri Desai^1^, Elena I. Queirolo^2^, Teresa Quattrin^3^, Patrick J. Parsons^4,5^, Christopher D. Palmer^4,5^, María Inés Beledo^2^, Katarzyna Kordas^1^

^1^*Department of Epidemiology and Environmental Health, University at Buffalo, Buffalo, NY, USA*

*^2^Department of Neuroscience and Learning, Catholic University of Uruguay, Montevideo, Uruguay*

*^3^Department of Pediatrics, Jacobs School of Medicine and Biomedical Sciences, University at Buffalo, Buffalo, NY, USA*

*^4^Division of Environmental Health Sciences, Wadsworth Center, New York State Department of Health, Albany, NY, USA*

*^5^Department of Environmental Health Sciences, College of Integrated Health Sciences, University at Albany, Albany, NY, USA*

**Table of contents**

| **Contents** | **Page no.** |
| --- | --- |
| Details of Analytical Methods | 2 |
| Supplemental Table 1: Inductively Coupled Plasma Tandem Mass Spectrometry (ICP-MS/MS) method validation against two National Institute of Standards and Technology (NIST) standard materials for blood arsenic (As) and lead (Pb) | 3 |
| Supplemental Table 2: Details of stable isotopes monitored by Inductively Coupled Plasma Tandem Mass Spectrometry (ICP-MS/MS) | 4 |
| Supplemental Table 3: Thermo Scientific iCAP^TM^ TQ operational parameters | 5 |
| Supplemental Table 4: Medians (ranges) of blood arsenic and serum lipid markers by characteristics of Uruguayan adolescents participating in the Complex Mixtures, Oxidative Stress, and Cognition Study | 6 |
| Supplemental Table 5: Sociodemographic, anthropometric, and biochemical characteristics of Uruguayan participants adolescents included in the complete case sample compared to those excluded from the sample | 7 |
| Supplemental Table 6: Mean (SD) lipid marker levels according to tertiles of averaged blood As levels among Uruguayan adolescents (n=327) | 8 |
| Supplemental Table 7: Associations between averaged, log-transformed blood arsenic concentrations and lipids stratified by body mass index percentiles among Uruguayan study participants | 9 |
| Supplemental Table 8: Associations between averaged, log-transformed blood arsenic concentrations and lipids stratified by sex among Uruguayan study participants | 10 |
| Supplemental Table 9: Association between blood arsenic concentrations^1^ and lipids among Uruguayan study participants with arsenic measures available at both visits (n=319) | 11 |

**Details of Analytical Methods**

Arsenic (As) and lead (Pb): Limits of detection (LODs) based on a blood matrix were calculated per IUPAC guidelines ranged from 0.045 to 0.088 µg/L for As and from 0.04 to 0.065 µg/dL for Pb across more than 20 analytical runs; none of the samples had values below the LOD. Three concentration levels of internal QC blood materials were analyzed at the start, end, and throughout each analytical run. Typical method repeatability (i.e., between-run precision or CV) for As was 3.8% at 0.49 µg/L; 2.8% at 2.1 µg/L; and 2.2% at 4.0 µg/L. For blood Pb, typical CVs were 3.4% at 3.0 µg/dL; 2.3% at 12 µg/dL; and 2.3% at 41 µg/dL.

Serum total cholesterol: Precision was verified using human samples and controls with reproducibility (n=21) and intermediate precision (3 aliquots per run, 1 run per day, 21 days) on the Cobas c 501 analyzer. Repeatability was found to show a mean of 2.85 mmol/L (110 mg/dL) [SD 0.03 mmol/L (1 mg/dL)] and 7.39 mmol/L (286 mg/dL) [SD 0.05 mmol/L (2 mg/dL)] for human serum 1 and 2, respectively. The coefficients of variance were 1.1% and 0.7%, respectively. The intermediate precision had a mean of 1.97 mmol/L (76.2 mg/dL) [SD 0.03 mmol/L (1.2 mg/dL)] and 7.13 mmol/L (276 mg/dL) [SD 0.10 mmol/L (4 mg/dL)] for human serum 3 and 4, respectively. The coefficients of variance were 1.6% and 1.4%, respectively. The lower limit of detection was 3.86 mg/dL and the day-to-day imprecision values at concentrations of 76.2 and 276 mg/dL were 1.6% and 1.4%, respectively.

Serum Triglycerides: Precision was verified using human samples and controls with reproducibility (n=21) and intermediate precision (3 aliquots per run, 1 run per day, 21 days) on the Cobas c 501 analyzer. Repeatability was found to show a mean of 1.67 mmol/L (148 mg/dL) [SD 0.02 mmol/L (2 mg/dL)] and 2.72 mmol/L (241 mg/dL) [SD 0.02 mmol/L (2 mg/dL)] for human serum 1 and 2, respectively. The coefficients of variance were 1.1% and 0.7%, respectively. The intermediate precision had a mean of 1.18 mmol/L (104 mg/dL) [SD 0.02 mmol/L (2 mg/dL)] and 2.95 mmol/L (261 mg/dL) [SD 0.05 mmol/L (4 mg/dL)] for human serum 3 and 4, respectively. The coefficients of variance were 1.9% and 1.8%, respectively. The lower limit of detection was 8.85 mg/dL and the day-to-day imprecision values at concentrations of 104 and 261 mg/dL were 1.9% and 1.8%, respectively.

Serum High Density Lipoprotein Cholesterol: Precision was verified on the Cobas c 501 analyzer using human samples and controls in accordance with the Clinical and Laboratory Standards Institute EP5 requirements (4 aliquots per run, 1 run per day, 21 days). The coefficients of variance for repeatability ranged from 0.6% to 1.8% and those for intermediate precision ranged from 0.7% to 2.2% for human serum samples 1-5. The lower limit of detection was 3.09 mg/dL and the day-to-day imprecision values at concentrations of 9.48, 59.4 and 141 mg/dL were 2.2%, 0.7% and 0.8%, respectively.

**Supplemental Table 1: Inductively Coupled Plasma Tandem Mass Spectrometry (ICP-MS/MS) method validation against two National Institute of Standards and Technology (NIST) standard materials for blood arsenic (As) and lead (Pb).**

|  | **As** | | | **Pb** | | |
| --- | --- | --- | --- | --- | --- | --- |
|  | **Certified value**  **± U^a^, µg/L As** | **NYS found value**  **± 95% confidence interval^b^, µg/L As** | **% Bias** | **Certified value**  **± U^a^, µg/dL Pb** | **NYS found value**  **± 95% confidence interval^b^, µg/dL Pb** | **% Bias** |
| **NIST 955c in Caprine Blood** | | | | | | |
| NIST 955c Level 1 | <5^c^ | 0.110 ± 0.009 | - | 0.424 ±0.011 | 0.416 ±0.010 | -1.9 |
| NIST 955c Level 2 | 21.66 ± 0.73 | 20.22 ± 0.12 | -6.6 | 13.950 ±0.080 | 13.71 ±0.11 | -1.7 |
| NIST 955c Level 3 | 52.7 ± 1.1 | 51.6 ± 0.3 | -2.1 | 27.76 ±0.16 | 27.33 ±0.18 | -1.5 |
| NIST 955c Level 4 | 78.8 ± 4.9 | 77.8 ± 0.7 | -1.3 | 45.53 ±0.27 | 44.92 ±0.49 | -1.3 |
| **NIST 955d Metals and Metabolites in Frozen Human Blood** | | | | | | |
| NIST 955d Level 1 | 5.31 ± 0.76 | 4.88 ± 0.14 | -8.1 | 1.48 ±0.026 | 1.43 ±0.02 | -3.4 |
| NIST 955d Level 2 | 277.5 ± 4.8 | 271 ± 3.2 | -2.3 | 4.947 ±0.085 | 4.80 ±0.02 | -2.9 |
| NIST 955d Level 3 | 774 ± 13 | 757 ± 0.32 | -2.2 | 42.13 ±0.63 | 41.30 ±0.34 | -2.0 |

^a^ NIST uncertainty expressed as the Guide to Measurement Uncertainty (GUM) expanded uncertainty, U, the 95% confidence level, reflecting the combined effects of measurement uncertainty, blanks, and any systematic differences between techniques when more than one method was used to assign a value.

^b^ Method imprecision estimated using found values and calculated as the 95% confidence interval from independent measurements (n=5) obtained during the June 2022 method validation which was performed immediately before the analysis of project samples.

^c^ NIST information value.

**Supplemental Table 2: Details of stable isotopes monitored by Inductively Coupled Plasma Tandem Mass Spectrometry (ICP-MS/MS).**

| **Analyte**  **Mass to Charge**  **(m/z)** | **Measured**  **Mass to Charge**  **(m/z)** | **Internal Standard**  **(mode)** | **Major Interferences on Analyte Mass to Charge**  **(% abundance listed in Qtegra^TM^ software)** | **Instrument Mode Applied to Resolve Polyatomic and Isobaric Interferences at the Measured Mass to Charge** |
| --- | --- | --- | --- | --- |
| ^75^As | 91  (^75^As^16^O^+^) | ^71^Ga^+^  (TQ-O_2_ on mass) | ^38^Ar^37^Cl^+^ (0.02); ^40^Ar^35^Cl^+^ (75.48); ^16^O^59^Co^+^ (99.76); ^12^C^63^Cu^+^ (68.41); ^16^O^1^H^58^Ni^+^ (68.1); ^1^H^74^Ge^+^ (36.50); ^14^N^61^Ni^+^ (1.13)  ^149^Sm^++^ (13.80); ^150^Sm^++^ (7,40); ^150^Nd^++^ (5.64) | TQ-O_2_ Gas Mode  mass-shift away from interferences  Q1: iMS filter (Normal)  Q3: 0.7 amu filter (Normal) |
| ^206,207,208^Pb | Sum of 206+207+208  (^206^Pb^+^ +^207^Pb^+^  +^208^Pb^+^) | ^193^Ir^+^  (KED) | ^206^Pb^+^:  ^1^H^205^Tl+ (70.47); ^14^H^192^Os^+^ (40.85); ^40^Ar^166^Er^+^ (33.47); ^12^C^194^Pt^+^ (32.54); ^16^O^190^Os^+^ (26.34); ^16^O^1^H^189^Os^+^ (16.06)  ^207^Pb^+^:  ^14^N^193^Ir^+^ (62.47); ^16^O^191^Ir^+^ (37.43); ^12^C^195^Pt^+^ (33.43); ^16^O^1^H^190^Os^+^ (26.33); ^1^H^206^Pb^+^ (24.10); ^40^Ar^167^Er^+^ (22.86)  ^208^Pb^+^:  ^16^O^192^Os^+^ (40.90); ^16^O^1^H^191^Ir^+^ (37.21); ^14^N^194^Pt^+^ (32.78); ^40^Ar^168^Er^+^ (26.69); ^12^C^196^Pt+ (25.02); ^1^H^207^Pb^+^ (22.10) | All three Pb isotopes:  SQ-KED Mode (He gas)  Q1: SQ  Q3: 0.7 amu filter (Normal) |
| *^71^Ga*  **Internal*  *standard* | 71  (^71^Ga^+^) | N/A | ^36^Ar^35^Cl^+^ (0.255); ^16^O^55^Mn^+^ (99.76); ^40^Ar^31^P^+^ (99.60); ^12^C^59^Co^+^ (98.90); ^1^H^70^Ge^+^ (20.50); ^16^O^1^H^54^Fe^+^ (5.79); ^16^O^1^H^54^Cr^+^ (2.60); ^14^N^57^Fe^+^ (2.19) | SQ-KED; TQ-O_2_/iO_2_ (on-mass);  TQ-He dependent on the target analyte.  Q1: iMS filter (Normal)  Q3: 0.7 amu filter (Normal) |
| *^193^Ir*  **Internal*  *standard* | 193  (^193^Ir^+^) | N/A | ^12^C^181^Ta^+^ (98.89); ^40^Ar^153^Eu^+^ (51.99); ^1^H^192^Os^+^ (40.99); ^16^O^177^Hf^+^ (18.56); ^14^N^179^Hf^+^ (13.58); ^16^O^1^H^176^Yb^+^ (12.67); ^16^O^1^H^176^Hf^+^ (5.19); ^16^O^1^H^176^Lu^+^ (2.58) | Used as the I.S. for ‘heavier’ analytes.  SQ-KED  TQ-He (W only)  Q1: iMS filter (Normal)  Q3: 0.7 amu filter (Normal) |

**Supplemental Table 3: Thermo Scientific iCAP^TM^ TQ operational parameters.**

| **Parameter** | **Typical ICP-MS/MS Setting** |
| --- | --- |
| Rf Power | 1000 – 1500 W |
| Ar Nebulizer Gas Flowrate | 0.4 – 1.2 L/min (dependent on Nebulizer type) |
| Sweeps/Reading | 90 |
| Readings/Replicate | 1 |
| Replicates | 3 |
| Dwell Time | 15 ms |
| Detector Mode | Dual (Pulse and Analogue) |
| Measurement Units | Counts per Second (cps) |
| Curve Type | Linear (Absolute or None weighting) |
| Units | **µg/L** – Be, V, Cr, Mn, Co, Ni, Cu, Zn, As, Se, Sr, Mo, Cd, Sn, Sb, Te, Cs, Ba, W, Pt, Hg, Tl  **µg/dL** – Pb |

**Supplemental Table 4: Medians (ranges) of blood arsenic and serum lipid markers by characteristics of Uruguayan adolescents participating in the Complex Mixtures, Oxidative Stress, and Cognition Study.**

| **Covariates^1^** | **N^2^** | **Blood As**  **Visit 1, µg/L** | **Average Blood As, µg/L** | **N^3^** | **Blood As**  **Visit 2, µg/L** | **Total cholesterol, mg/dL** | **HDL cholesterol, mg/dL** | **Non-HDL cholesterol, mg/dL** | **Triglycerides, mg/dL** |
| --- | --- | --- | --- | --- | --- | --- | --- | --- | --- |
| Age, years  <11.6  ≥11.6 | 151  168 | 0.37 (0.18, 4.01)  0.40 (0.20, 2.15) | 0.37 (0.20, 2.54)  0.39 (0.20, 2.22) | 156  171 | 0.36 (0.16, 4.81)  0.36 (0.14, 4.11) | 155 (104, 261)  151 (81.0, 302) | 50.0 (21.0, 83.0)  48.0 (26.0, 93.0) | 102 (55.0, 178)  100 (42.0, 260) | 71.0 (21.0, 235)  67.0 (27.0, 291) |
| Sex  Girls, n (%)  Boys, n (%) | 161  158 | 0.39 (0.18, 4.01)  0.38 (0.20, 3.04) | 0.37 (0.20, 2.54)  0.40 (0.20, 2.22) | 165  162 | 0.34 (0.14, 4.81)  0.37 (0.16, 4.11) | 150 (101, 302)  156 (81.0, 234) | 49.0 (26.0, 84.0)  48.5 (21.0, 93.0) | 100 (61.0, 260)  102 (42.0, 176) | 74.0 (25.0, 261)  65.0 (21.0, 291) |
| BMI, kg/m^2^  <20.3  ≥20.3 | 157  162 | 0.37 (0.18, 4.01)  0.40 (0.21, 3.80) | 0.37 (0.20, 2.22)  0.40 (0.20, 2.54) | 160  167 | 0.35 (0.16, 4.11)  0.37 (0.14, 4.81) | 154 (81.0, 261)  151 (99.0, 302) | 52.5 (21.0, 84.0)  46.0 (26.0, 93.0) | 99.5 (42.0, 178)  103 (54.0, 260) | 64.0 (21.0, 181)  75.0 (30.0, 291) |
| Secondhand Smoke Exposure  None  Low  High | 128  125  66 | 0.42 (0.20, 4.01)  0.38 (0.18, 2.18)  0.36 (0.18, 3.80) | 0.40 (0.20, 2.20)  0.37 (0.22, 2.22)  0.37 (0.22, 2.54) | 129  129  69 | 0.36 (0.17, 1.31)  0.36 (0.14, 4.11)  0.35 (0.16, 4.81) | 148 (100, 261)  152 (81.0, 302)  156 (99.0, 222) | 48.0 (26.0, 93.0)  49.0 (28.0, 76.0)  48.0 (21.0, 82.0) | 100 (54.0, 178)  101 (49.0, 260)  104 (42.0, 156) | 65.0 (25.0, 291)  71.0 (21.0, 261)  72.0 (28.0, 203) |
| Physical Activity Score  <1.65  ≥1.65 | 168  151 | 0.38 (0.18, 4.01)  0.40 (0.18, 3.04) | 0.38 (0.21, 2.22)  0.39 (0.20, 2.54) | 175  152 | 0.35 (0.14, 4.11)  0.37 (0.17, 4.81) | 154 (81.0, 302)  152 (99.0, 234) | 48.0 (21.0, 83.0)  49.5 (34.0, 93.0) | 102 (42.0, 260)  101 (54.0, 172) | 71.0 (27.0, 291)  65.0 (21.0, 260) |
| Household Assets Score  <0.18  ≥0.18 | 156  163 | 0.39 (0.20, 3.80)  0.38 (0.18, 4.01) | 0.39 (0.20, 2.54)  0.38 (0.20, 2.20) | 163  164 | 0.38 (0.14, 4.81)  0.35 (0.17, 1.31) | 154 (99.0, 302)  153 (81.0, 261) | 47.0 (21.0, 84.0)  50.0 (26.0, 93.0) | 100 (42.0, 260)  103 (49.0, 194) | 69.0 (21.0, 291)  70.0 (27.0, 260) |
| Average blood Pb, µg/dL  <1.13  ≥1.13 | 158  161 | 0.38 (0.20, 4.01)  0.39 (0.18, 2.18) | 0.39 (0.20, 2.54)  0.38 (0.20, 2.22) | 163  164 | 0.35 (0.14, 4.81)  0.37 (0.16, 4.11) | 151 (101, 261)  155 (81.0, 302) | 50.0 (26.0, 93.0)  48.0 (21.0, 82.0) | 100 (42.0, 194)  102 (49.0, 260) | 71.0 (21.0, 260)  67.5 (25.0, 291) |

^1^Continuous variables are split at the median

^2^The sample size applies to blood As at visit 1 and average blood As

^3^The sample size applies to blood As at visit 2 and serum lipid markers

**Supplemental Table 5: Sociodemographic, anthropometric, and biochemical characteristics of Uruguayan participants adolescents included in the complete case sample compared to those excluded from the sample.**

|  | **Included in the complete case sample** | | **Excluded from the complete case sample** | |
| --- | --- | --- | --- | --- |
| **Variables** | **N** | **Value** | **N** | **Value** |
| Age, years  Median (range) | 327 | 11.7 (8.59, 19.8) | 103 | 11.9 (8.36, 20.3) |
| Sex  Girls, n (%)  Boys, n (%) | 165  162 | 165 (50.5)  162 (49.4) | 51  43 | 51 (54.3)  43 (45.7) |
| Blood As at time 1, µg/L  Median (range) | 319 | 0.38 (0.18, 4.01) | 85 | 0.36 (0.19, 1.75) |
| Blood As at time 2, µg/L  Median (range) | 327 | 0.36 (0.14, 4.81) | 51 | 0.38 (0.17, 1.74) |
| Blood Pb at time 1, µg/dL  Median (range) | 319 | 1.17 (0.28, 43.2) | 86 | 1.25 (0.44, 17.5) |
| Blood Pb at time 2, µg/dL  Median (range) | 327 | 1.03 (0.39, 24.8) | 51 | 1.13 (0.36, 3.30) |
| BMI, kg/m^2^  Median (range) | 327 | 20.3 (13.0, 43.5) | 72 | 21.2 (14.1, 34.5) |
| Secondhand Smoke Exposure  None  Low  High | 129  129  69 | 129 (39.5)  129 (39.5)  69.0 (21.1) | 35  54  13 | 35 (34.3)  54 (52.9)  13 (12.8) |
| Physical Activity Score  Median (range) | 327 | 1.64 (0.94, 2.59) | 102 | 1.53 (1.00, 2.56) |
| Household Assets Score  Median (range) | 327 | 0.18 (-2.26, 1.69) | 100 | 0.24 (-2.24, 1.77) |
| Total cholesterol, mg/dL  Median (range) | 327 | 153 (81.0, 302) | 45 | 144 (89.0, 209) |
| HDL cholesterol, mg/dL  Median (range) | 327 | 49.0 (21.0, 93.0) | 45 | 46.0 (25.0, 75.0) |
| Non-HDL cholesterol, mg/dL  Median (range) | 327 | 101 (42.0, 260) | 45 | 100 (44.0, 160) |
| Triglycerides, mg/dL  Median (range) | 327 | 70.0 (21.0, 291) | 45 | 70.0 (36.0, 260) |

**Supplemental Table 6: Mean (SD) lipid marker levels according to tertiles of averaged blood As levels among Uruguayan adolescents (n=327).**

|  | **Averaged Blood As levels**, µg/L | | |
| --- | --- | --- | --- |
|  | **Tertile 1**  (≤0.34) | **Tertile 2**  (>0.34 and ≤0.425) | **Tertile 3**  (>0.425) |
| Total cholesterol, mg/dL | 150 (26.5) | 156 (27.0) | 156 (32.7) |
| HDL cholesterol, mg/dL | 49.5 (10.6) | 51.4 (10.8) | 49.4 (11.1) |
| Non-HDL cholesterol, mg/dL | 101 (23.0) | 105 (26.6) | 107 (31.0) |
| Triglycerides, mg/dL | 76.2 (33.2) | 80.0 (43.3) | 87.5 (51.6) |

**Supplemental Table 7: Associations between averaged, log-transformed blood arsenic concentrations and lipids stratified by body mass index percentiles among Uruguayan study participants.**

|  | **Complete case dataset (n=327)** | | | | **Imputed dataset (n=337)** | | | |
| --- | --- | --- | --- | --- | --- | --- | --- | --- |
|  | **BMI < 85^th^ percentile**  **(n=187)** | | **BMI ≥ 85^th^ percentile**  **(n=140)** | | **BMI < 85^th^ percentile**  **(n=192)** | | **BMI ≥ 85^th^ percentile**  **(n=145)** | |
|  | **Crude β**  **(95% CI)** | **Adjusted^1^ β (95% CI)** | **Crude β**  **(95% CI)** | **Adjusted^1^ β (95% CI)** | **Crude β**  **(95% CI)** | **Adjusted^1^ β (95% CI)** | **Crude β**  **(95% CI)** | **Adjusted^1^ β (95% CI)** |
| Total cholesterol | 6.55  (-3.65, 16.8) | 8.11  (-2.28, 18.5) | 7.46  (-4.84, 19.8) | 2.37  (-9.99, 14.7) | 6.75  (-3.34, 16.8) | 8.30  (-1.94, 18.5) | 4.35  (-7.22, 15.9) | -0.81  (-12.4, 10.8) |
| HDL cholesterol | 2.24  (-1.67, 6.15) | 3.01  (-0.92, 6.95) | -3.10  (-7.29, 1.09) | -2.84  (-7.21, 1.52) | 2.54  (-1.32, 6.39) | 3.23  (-0.65, 7.10) | -3.61  (-7.68, 0.45)^#^ | -3.79  (-8.02, 0.43)^#^ |
| Non-HDL cholesterol | 4.31  (-4.82, 13.4) | 5.10  (-4.25, 14.5) | 10.6  (-1.26, 22.4) | 5.21  (-6.69, 17.1) | 4.21  (-4.81, 13.24) | 5.07  (-4.15, 14.3) | 7.97  (-3.08, 19.0) | 2.98  (-8.14, 14.1) |
| Triglycerides | -4.80  (-16.2, 6.59) | -3.79  (-15.3, 7.72) | 34.4  (13.3, 55.4)^*^ | 30.3  (8.24, 52.4)^*^ | -4.34  (-15.5, 6.86) | -3.20  (-14.5, 8.14) | 27.1  (7.35, 47.0)^*^ | 23.9  (3.17, 44.7)^*^ |

*Abbreviation: BMI: body mass index; HDL: high-density lipoprotein*

*^1^Adjusted for sex, age, secondhand smoke exposure, physical activity score, household assets score, and average blood lead levels from the two measures (one measure if two were unavailable)*

*^*^p<0.05*

*^#^p<0.1*

**Supplemental Table 8: Associations between averaged, log-transformed blood arsenic concentrations and lipids stratified by sex among Uruguayan study participants.**

|  | **Complete case dataset (n=327)** | | | | **Imputed dataset (n=337)** | | | |
| --- | --- | --- | --- | --- | --- | --- | --- | --- |
|  | **Girls (n=165)** | | **Boys (n=162)** | | **Girls (n=174)** | | **Boys (n=163)** | |
|  | **Crude β**  **(95% CI)** | **Adjusted^1^ β (95% CI)** | **Crude β**  **(95% CI)** | **Adjusted^1^ β (95% CI)** | **Crude β**  **(95% CI)** | **Adjusted^1^ β (95% CI)** | **Crude β**  **(95% CI)** | **Adjusted^1^ β (95% CI)** |
| Total cholesterol | 15.0  (4.62, 25.5)^*^ | 12.4  (2.13, 22.6)^*^ | -2.81  (-14.6, 8.93) | -1.89  (-14.1, 10.3) | 13.9  (3.57, 24.2)^*^ | 10.8  (0.63, 21.0)^*^ | -3.88  (-15.2, 7.41) | -2.66  (-14.6, 9.23) |
| HDL cholesterol | -0.86  (-4.74, 3.02) | 0.38  (-3.37, 4.13) | 0.19  (-4.36, 4.74) | 1.06  (-3.47, 5.59) | -1.10  (-5.06, 2.86) | -0.59  (-4.64, 3.46) | -0.23  (-4.82, 4.35) | 0.52  (-4.28, 5.31) |
| Non-HDL cholesterol | 15.9  (6.42, 25.4)^*^ | 12.0  (2.81, 21.1)^*^ | -3.00  (-14.2, 8.21) | -2.95  (-14.5, 8.63) | 15.0  (5.63, 24.3)^*^ | 11.4  (2.38, 20.5)^*^ | -3.64  (-14.4, 7.08) | -3.17  (-14.4, 8.05) |
| Triglycerides | 26.6  (11.1, 42.1)^*^ | 20.3  (5.17, 35.4)^*^ | -2.20  (-19.6, 15.2) | -4.25  (-21.1, 12.6) | 25.0  (9.72, 40.3)^*^ | 20.5  (5.39, 35.7)^*^ | -3.21  (-19.9, 13.5) | -4.31  (-21.7, 13.1) |

*Abbreviation: HDL: high-density lipoprotein*

*^1^Adjusted for age, body mass index, secondhand smoke exposure, physical activity score, household assets score, and average blood lead levels from the two measures (one measure if two were unavailable)*

*^*^p<0.05*

**Supplemental Table 9:** **Association between blood arsenic concentrations^1^ and lipids among Uruguayan study participants with arsenic measures available at both visits (n=319).**

|  | **Crude β (95% CI)** | **Adjusted^2^ β (95% CI)** |
| --- | --- | --- |
| Total cholesterol | 7.69 (-0.22, 15.6)^#^ | 7.27 (-0.76, 15.3)^#^ |
| HDL cholesterol | 0.34 (-2.61, 3.28) | 0.77 (-2.10, 3.64) |
| Non-HDL cholesterol | 7.35 (-0.05, 14.8)^#^ | 6.50 (-0.96, 14.0) |
| Triglycerides | 12.4 (0.58, 24.2)^*^ | 11.1 (-0.35, 22.5)^#^ |

*Abbreviation: HDL: high-density lipoprotein*

*^1^Blood As was measured 1 year before and concurrently to the endpoint; it was averaged, log-transformed for analysis.*

*^2^Adjusted for sex, age, body mass index, secondhand smoke exposure, physical activity score, household assets score, and average blood lead levels from the two measures*

*^*^p<0.05*

*^#^p<0.1*
